# Quantitative imaging biomarkers of coronary plaque morphology: insights from EVAPORATE

**DOI:** 10.3389/fcvm.2023.1204071

**Published:** 2023-08-03

**Authors:** Andrew J. Buckler, Gheorghe Doros, April Kinninger, Suvasini Lakshmanan, Viet T. Le, Peter Libby, Heidi T. May, Joseph B. Muhlestein, John R. Nelson, Anna Nicolaou, Sion K. Roy, Kashif Shaikh, Chandana Shekar, John A. Tayek, Luke Zheng, Deepak L. Bhatt, Matthew J. Budoff

**Affiliations:** ^1^ Department of Molecular Medicine, Karolinska Institutet, Stockholm, Sweden; ^2^ Elucid Bioimaging Inc., Boston, MA, United States; ^3^ BAIM Institute, Boston, MA, United States; ^4^ Department of Medicine, Lundquist Institute at Harbor-UCLA Medical Center, Torrance, CA, United States; ^5^ Intermountain Heart Institute, Intermountain Medical Center, Salt Lake City, UT, United States; ^6^ Rocky Mountain University of Health Profession, Provo, UT, United States; ^7^ Brigham and Women’s Hospital Heart & Vascular Center and Harvard Medical School, Boston, MA, United States; ^8^ California Cardiovascular Institute, Fresno, CA, United States; ^9^ Mount Sinai Heart, Icahn School of Medicine at Mount Sinai Health System, New York, NY, United States

**Keywords:** atherosclerosis, biomarker, plaque, CTA, lipidemia

## Abstract

**Aims:**

Residual cardiovascular risk persists despite statin therapy. In REDUCE-IT, icosapent ethyl (IPE) reduced total events, but the mechanisms of benefit are not fully understood. EVAPORATE evaluated the effects of IPE on plaque characteristics by coronary computed tomography angiography (CCTA). Given the conclusion that the IPE-treated patients demonstrate that plaque burden decreases has already been published in the primary study analysis, we aimed to demonstrate whether the use of an analytic technique defined and validated in histological terms could extend the primary study in terms of whether such changes could be reliably seen in less time on drug, at the individual (rather than only at the cohort) level, or both, as neither of these were established by the primary study result.

**Methods and Results:**

EVAPORATE randomized the patients to IPE 4 g/day or placebo. Plaque morphology, including lipid-rich necrotic core (LRNC), fibrous cap thickness, and intraplaque hemorrhage (IPH), was assessed using the ElucidVivo® (Elucid Bioimaging Inc.) on CCTA. The changes in plaque morphology between the treatment groups were analyzed. A neural network to predict treatment assignment was used to infer patient representation that encodes significant morphological changes. Fifty-five patients completed the 18-month visit in EVAPORATE with interpretable images at each of the three time points. The decrease of LRNC between the patients on IPE vs. placebo at 9 months (reduction of 2 mm^3^ vs. an increase of 41 mm^3^, *p* = 0.008), widening at 18 months (6 mm^3^ vs. 58 mm^3^ increase, *p* = 0.015) were observed. While not statistically significant on a univariable basis, reductions in wall thickness and increases in cap thickness motivated multivariable modeling on an individual patient basis. The per-patient response assessment was possible using a multivariable model of lipid-rich phenotype at the 9-month follow-up, *p* < 0.01 (sustained at 18 months), generalizing well to a validation cohort.

**Conclusion:**

Plaques in the IPE-treated patients acquired more characteristics of stability. Reliable assessment using histologically validated analysis of individual response is possible at 9 months, with sustained stabilization at 18 months, providing a quantitative basis to elucidate drug mechanism and assess individual patient response.

## 1. Introduction

In the Reduction of Cardiovascular Events with EPA—Intervention Trial (REDUCE-IT) , icosapent ethyl (IPE) yielded a reduction of 25% in major cardiovascular (CV) events and 32% in total events (1–8). IPE is the ethyl ester of eicosapentaenoic acid (EPA). This study quantitatively analyzed the effect of IPE on plaque morphology and composition in the patients enrolled in the Effect of Vascepa on Improving Coronary Atherosclerosis in People with High Triglycerides Taking Statin Therapy (EVAPORATE, ClinicalTrials.gov Identifier: NCT02926027). The EVAPORATE trial evaluated the effects of IPE on adverse atherosclerotic plaque characteristics by CCTA. The EVAPORATE assessed the change in low-attenuation plaque (LAP) volume by multidetector computed tomography angiography in 80 statin-treated patients randomized to 4 g/day IPE or placebo at 9 and 18 months (9–11). The prespecified primary endpoint of change in LAP volume was met at 18 months between the IPE and placebo groups.

Given that LAP lacks an objective definition, our study aimed to compare this result with what could be done using objectively defined tissue markers such as histologically defined and validated lipid-rich necrotic core (LRNC). Specifically, this study used a software validated using histologic assessment to provide specific tissue characterization meeting requirements to be considered a biomarker, which enables granular mechanistic insight underlying the primary and secondary endpoints (12) using objective interpretation techniques (13–24). The purpose of this was twofold: first, to determine whether significant changes could be assessed earlier than with prior analytical methods, namely, at 9 months rather than needing 18; and second, whether an individualized patient model could be created for reliable classification of response at an individual level rather than only being significant at a cohort level (25).

## 2. Methods

The EVAPORATE trial randomized the statin-treated patients with high TG (135–499 mg/dl), well-controlled LDL, and known atherosclerosis to IPE 4 g/day or placebo. The plaque morphological characteristics, including LRNC, fibrous cap thickness (distance from the lumen to LRNC), and intraplaque hemorrhage (IPH), were assessed using ElucidVivo® (Elucid Bioimaging Inc., Boston, MA, USA) on CCTA. The per-patient multivariable predictive models discriminate the relevant mechanistic changes while allowing for individual physiological variation used to evaluate plaque morphology and provide a per-patient assessment tool. Specifically, multivariable modeling of LRNC together with wall and cap thickness was applied. The study endpoints, population, and design are as previously described (9).

**Figure 1:**
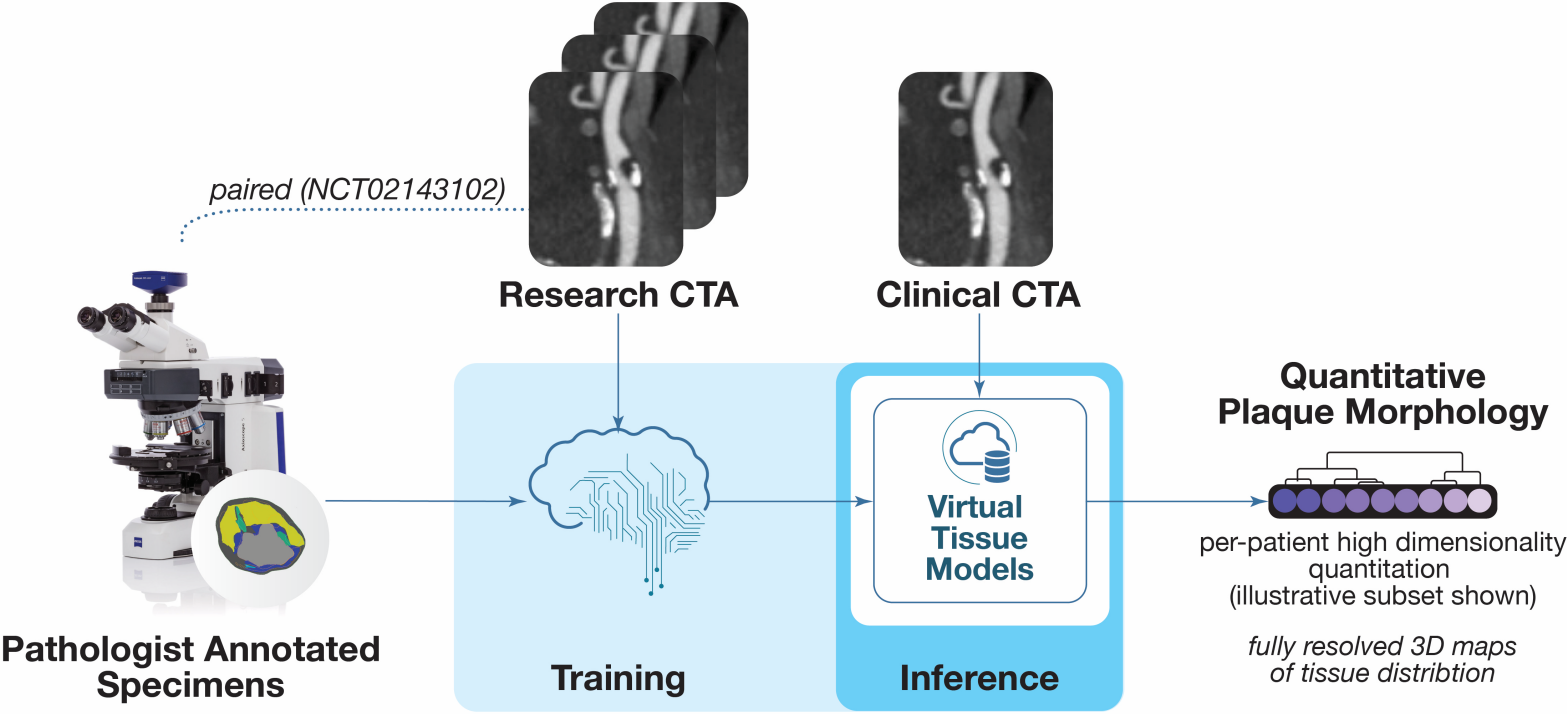
Overview of plaque analysis. The software analysis technique employs methods for deriving objective quantitative morphological assessment of structural anatomy and tissue characteristics from histopathology. This figure is a portion of an overview figure published initially in (46).

Coronary plaque analysis used commercially available software featuring a novel method for delineating the composition of vascular plaque components validated by expert-annotated histology ElucidVivo® (13–24) to extract quantitative plaque morphology comprising anatomic structure as well as tissue characteristics (Figure 1).

In the analytical method used for this study, the tissues are characterized according to strict definitions based on biological evidence on histopathology: LRNC, calcification (CALC), IPH, matrix (MATX), perivascular adipose tissue (PVAT), cap thickness (the smallest distance from LRNC to the lumen), and structural anatomic measurements such as degree of stenosis. LRNC is objectively defined as the accumulation of lipids by intimal/medial cells leading to progressive cell loss, cell death, degeneration, and necrosis. LRNC is a mixture of lipid, cellular debris, blood, water in various concentrations, lipid droplets intermixed extracellular matrix, necrotic amorphous eosinophilic material and is acellular, often surrounded by fibrotic tissue generated by smooth muscle cells/fibroblasts, and without microvasculature. CALC is a biological process that may stabilize plaque in some forms, and has a mechanism akin to bone formation, is observed as intimal/medial spaces with evidence of calcium primarily in the form of hydroxyapatite, osteoblasts or osteoid present, and no appreciable lipid or necrotic tissue. IPH is the accumulation of erythrocytes in the deeper regions of the plaque, with or without communication to the lumen or neovasculature, marked as erythrocytes, often in the deeper regions of the plaque. Fresh IPH is characterized by red blood cells (RBCs), intact and unorganized, whereas recent (5+ days) is observed as an inflammatory response with organized RBCs via hemolysis, fibroblast activity, and macrophage activity. MATX is the organization of macromolecules (such as collagen, elastin, glycoproteins, and proteoglycans) that provide structural support, tensile strength, or elasticity to the arterial wall, is observed as an intimal meshwork of dense or loose, homogeneous/organized collagen ECM (appear striated), embedded smooth muscle cells/fibroblasts (note elongated nuclei), and may have microvasculature.

*Statistical analyses*: Demographic and baseline characteristics were compared between the two treatment groups using the Fisher's exact test for categorical variables and the Wilcoxon rank-sum test for continuous variables. The variables were generally well-balanced between the two cohorts with minor exceptions. The changes from baseline to 9- and 18-month measurements were analyzed for each morphological characteristic to evaluate differences between the two groups. Student’s *t*-test was used to compare group means. Linear regression models were used to assess how baseline morphology changes differ across the two arms while adjusting for the effects of the variable EPA that showed an imbalance in the baseline comparisons of the arms. The models were adjusted by age, sex, diabetes, hypertension, and baseline triglyceride levels (26).

To extend the cohort result down to the individual patient level, we applied multivariable predictive modeling methods to mitigate per-patient physiological variations within and across arteries and elucidate the effect of the drug reliably for each individual patient. A quantitative analysis of the LRNC change, particularly when assessed with concomitant changes in other morphological characteristics (wall and cap thickness), can identify specific responses to the drug agents. The multivariable combination of all the morphological changes from baseline was used to derive the individual patient representation, encoding all the significant information of morphological change, using supervised machine learning classifiers to predict the correct treatment assignment. The data were partitioned to train (70%) and validation test (30%) sets in a stratified manner by making random splits in each treatment arm. All modeling was implemented using the Caret package in R. Unacceptably high correlations (>0.8) were identified in a heatmap where a clustering algorithm determines the order of columns and rows. A range of models (including averaged neural networks, support vector machines, and penalized logistic regression, with and without recursive feature elimination) were trained using the 10-fold cross-validation resampling scheme. They were optimized for the area under the receiver operating characteristic curve (AUROC) and Kappa (which adjusts for unbalanced covariates). The differing sets of morphological measurements according to hypothesized physiological rationale confirmed by an unsupervised clustering were used. The predictors were mean-centered, scaled to unit variance, and Yeo–Johnson transformed (27) to place the data on a scale where the distribution is approximately symmetric. These pre-processing steps are streamlined by Caret in the model-building process and contacted within the resampling steps. A true hold-out validation test set was sequestered to assess the ability of the models to generalize to unseen data.

## 3. Results

**Figure 2:**
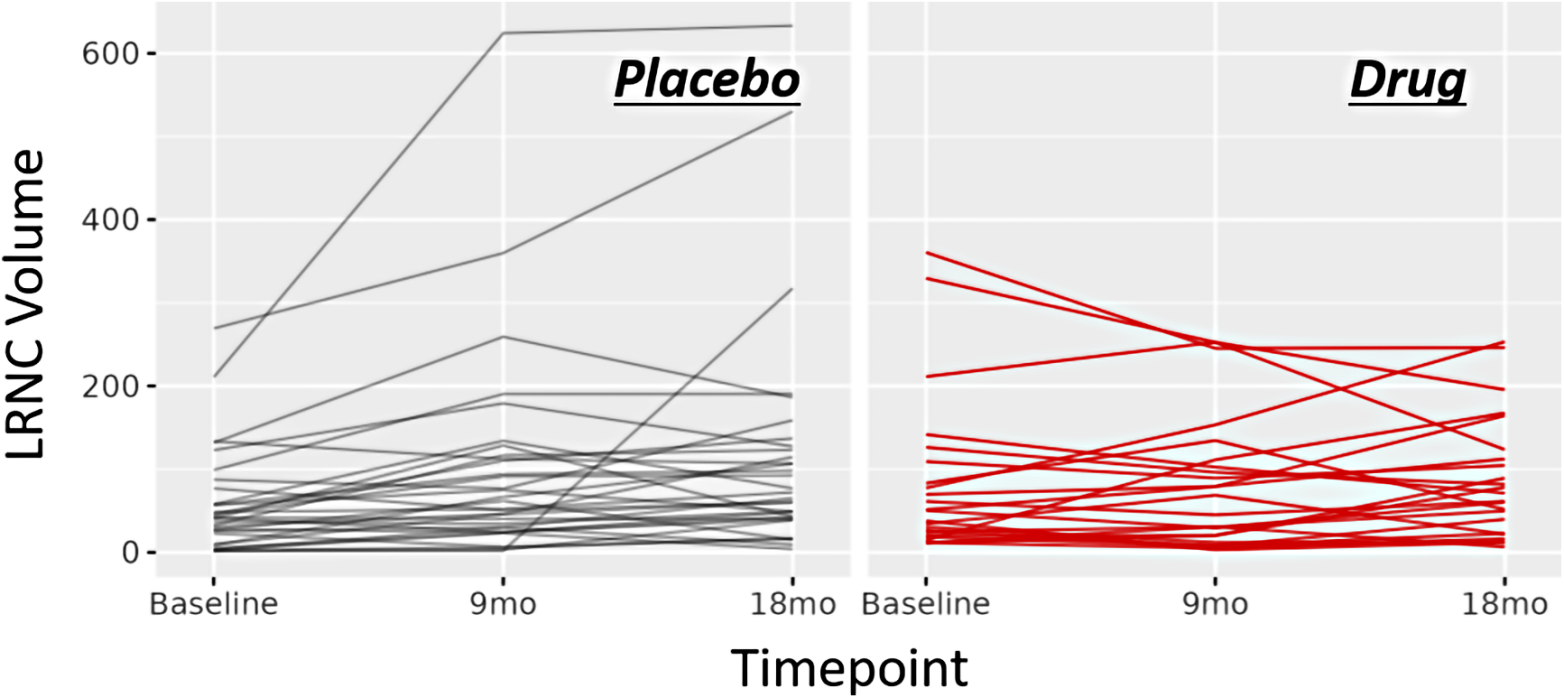
Line charts of morphology response. Line chart for change in LRNC volume across time points for the study cohort. Left: changes for the patients randomized to placebo. Right: changes for the patients randomized to drug.

**Figure 3:**
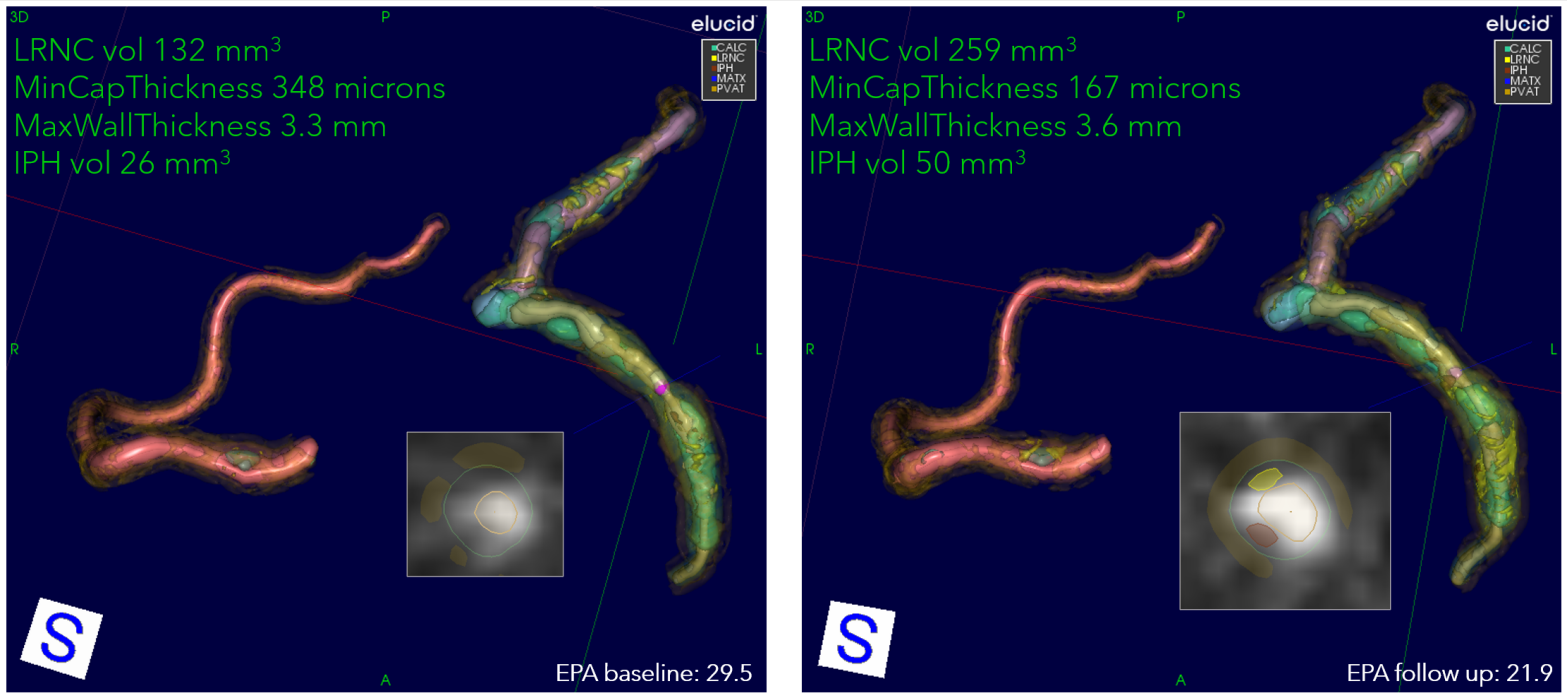
An example progression of patient on placebo. Overview of quantitation at two encounters of a patient on placebo: 72-year-old Asian female, baseline serum EPA 29.5, decreasing to 21.9 at 9 months. The 3D view of RCA, LAD, and LCX, with corner annotations for left coronary. The baseline values for LRNC volume (shown in yellow), minimum cap thickness, maximum wall thickness, and IPH volume (shown in rust) are 132 mm^3^, 348 μm, 3.3 mm, and 26 mm^3^, respectively. At follow-up, LRNC increased to 259 mm^3^, IPH to 50 mm^3^, cap thickness decreased to 167 μm, and wall thickness increased to 3.6 mm. The presentation of LRNC indicates coalescing of smaller lipid pools into larger contiguous cores at follow-up in all primary arteries. The 2D cross-section located at cursors in 3D representation shown as insets. Serum EPA values for baseline and follow-up are also shown.

**Figure 4:**
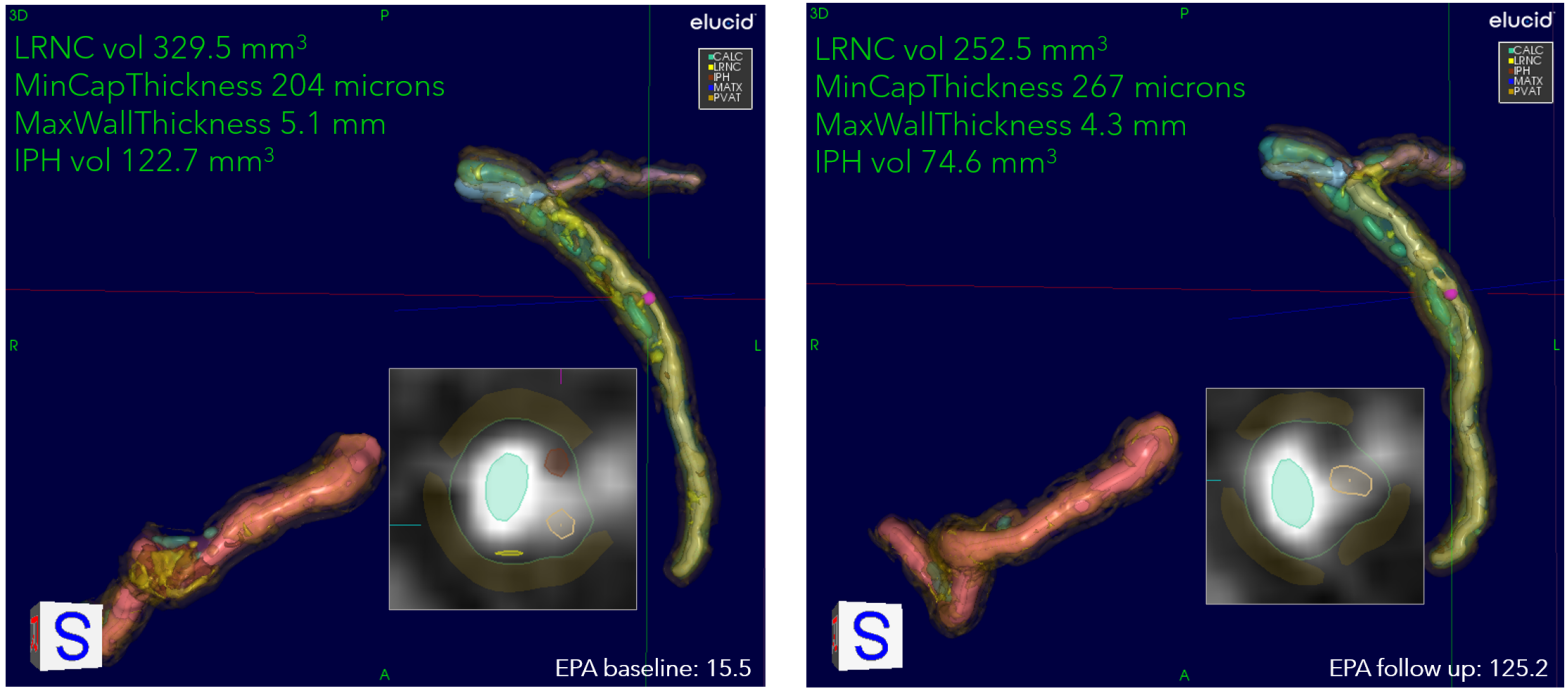
An example of stabilization/regression of patient on IPE. Overview of quantitation at two encounters of a patient on drug: 53-year-old White female, baseline serum EPA 15.5, increasing to 125.2 at the 9-month follow-up. The 3D view of RCA, LAD, and LCX, with corner annotations for left coronary. The baseline values for LRNC volume, minimum cap thickness, maximum wall thickness, and IPH volume are 329.5 mm^3^, 204 μm, 5.1 mm, and 122.7 mm^3,^ respectively. At follow-up, LRNC decreased to 252.5 mm^3^, IPH to 74.6 mm^3^, cap thickness increased to 267 μm, and wall thickness decreased to 4.3 mm. The presentation of residual LRNC indicates splitting into smaller lipid pools from larger contiguous cores at baseline. The 2D cross-section located at cursors in 3D representation shown as insets. Serum EPA values for baseline and follow-up are also shown.

Fifty-five patients completed the 18-month visit in EVAPORATE with interpretable images at each of the three time points (baseline, 9-, and 18-month follow-up). Table 1 shows the summary statistics of demographic and baseline characteristics stratified by the treatment group. The results are presented as counts and frequencies for the discrete variables and medians with interquartile ranges for the continuous variables. Table 2 shows unadjusted and adjusted *p*-values to detect changes at 9 and 18 months from baseline in morphological characteristics between the drug/placebo groups, reported from Student's *t*-test and linear regression models, respectively. The change in LRNC increased among those on placebo and initially decreased among those on IPE, but then modestly grew from 9 to 18 months, which yielded a large net difference in favor of IPE (Figure 2). The change of IPH in the treatment arm was small relative to LRNC, with a slight increase initially by 9 months and returning to baseline levels by 18 months. The maximum wall thickness increased for those randomized to placebo and decreased among those randomized to IPE. The fibrous cap thickness decreased among those taking placebo and increased among those receiving IPE. PVAT showed a similar pattern to IPH. The change in calcified volume increased for both arms but at a slower rate for those on IPE. Stenosis was equivocal across arms and time points. An example of a patient on placebo is given in Figure 3, and a patient on drug in Figure 4.

**Table 1:** Patient baseline characteristics.

Categorical variables	Baseline			Continuous variables	Baseline		
	Placebo	Drug	*p*-value		Placebo	Drug	*p*-value
White	75% (24/32)	87% (20/23)	0.33	Age	57.67 (11.40)	55.65 (10.34)	0.18
Male	53% (17/32)	57% (13/23)	1.00				
Chest pain	31% (10/32)	9% (2/23)	0.06	EPA	25.05 (14.57)	16.90 (11.40)	0.05
Menopause	93% (14/15)	60% (6/10)	0.12	Phosphorus	3.35 (0.58)	3.20 (0.50)	0.06
Hyperlipid meds	100% (32/32)	91% (21/23)	0.17	Lp_a	15.00 (2.75)	15.00 (34.00)	0.08
Aspirin	59% (19/32)	39% (9/23)	0.18	GSP	296 (170)	242 (125)	0.10
Angiogram	22% (7/32)	9% (2/23)	0.28	Mscl_CK	121 (103)	95 (53)	0.10
Kidney disease	3% (1/32)	13% (3/23)	0.30	BetaSitosterol	157 (164)	131 (103)	0.12
Angioplasty	12% (4/32)	4% (1/23)	0.39	Mscl_NTproBNP	43 (42)	57 (64)	0.16
Smoked past	88% (14/16)	100% (11/11)	0.50	Omega3FAIndex	2.02 (1.66)	1.89 (0.98)	0.22
Heart attack	3% (1/32)	9% (2/23)	0.57	eGFR	91 (29)	97 (21)	0.23
Lung disease	9% (3/32)	4% (1/23)	0.63	LDL	71 (59)	98 (48)	0.36
Hypertension	75% (24/32)	70% (16/23)	0.76	Triglycerides	199 (86)	194 (85)	0.51
Hypertension meds	78% (25/32)	74% (17/23)	0.76	Cholesterol	137 (63)	155 (48)	0.59
Diabetic	69% (22/32)	65% (15/23)	1.00	BMI	32.45 (8.55)	31.50 (8.45)	0.64
Diabetic meds	69% (22/32)	65% (15/23)	1.00	VLDL	27.5 (16)	28 (19)	0.85
FHX	34% (11/32)	30% (7/23)	1.00	HDL cholesterol	36.50 (13.50)	36.00 (12.00)	0.96

**Table 2:** Morphological changes by the treatment group.

Morphological characteristic	Baseline			Change at the 9-month visit					Change at the 18-month visit		
	Placebo	Drug	*p*-value	Placebo	Drug	*p*-value	Adj. *p*-value^a^	Adj. *p*-value^b^	Placebo	Drug	*p*-value
LRNC volume	83	56	0.25	41.1	−1.6	0.0	0.03	0.01	58	6	0.03
LRNC volume proportion	0.1	0.0	0.24	0.0	0.0	0.0	0.02	0.00	0.0	0.0	0.03
Max LRNC area proportion	0.4	0.4	0.19	0.1	0.0	0.1	0.14	0.10	0.1	0.0	0.13
PVAT volume proportion	1.5	1.6	0.56	−0.1	0.1	0.1	0.12	0.10	−0.1	0.1	0.21
Min cap thickness	0.3	0.4	0.08	0.0	0.0	0.2	0.20	0.30	0.0	0.0	0.15
Wall volume	1,595	1,543	0.63	93	16	0.2	0.28	0.41	109	60	0.51
Max IPH area	2.8	2.3	0.68	0.1	0.6	0.6	0.64	0.76	0.7	0.8	0.90
Max wall thickness	3.1	2.7	0.17	0.1	0.0	0.6	0.67	0.84	0.2	−0.1	0.18
Max diameter stenosis	0.6	0.7	0.14	0.0	0.0	0.6	0.91	0.74	0.0	0.0	0.51
IPH volume proportion	0.0	0.0	0.69	0.0	0.0	0.7	0.76	0.82	0.0	0.0	0.74
Plaque burden by volume	1.4	1.4	0.44	0.1	0.0	0.5	0.45	0.27	0.1	0.0	0.29
CALC volume	87	117	0.36	32	9	0.3	0.47	0.52	29	17	0.63
IPH volume	10.9	8.6	0.69	3.1	3.7	0.9	0.82	0.89	6.5	4.8	0.78

^a^ *p*-value adjusted for the baseline unbalanced variable EPA.

^b^ *p*-value adjusted for FA_EPA, age, sex, diabetes, hypertension, and baseline triglycerides.

**Figure 5:**
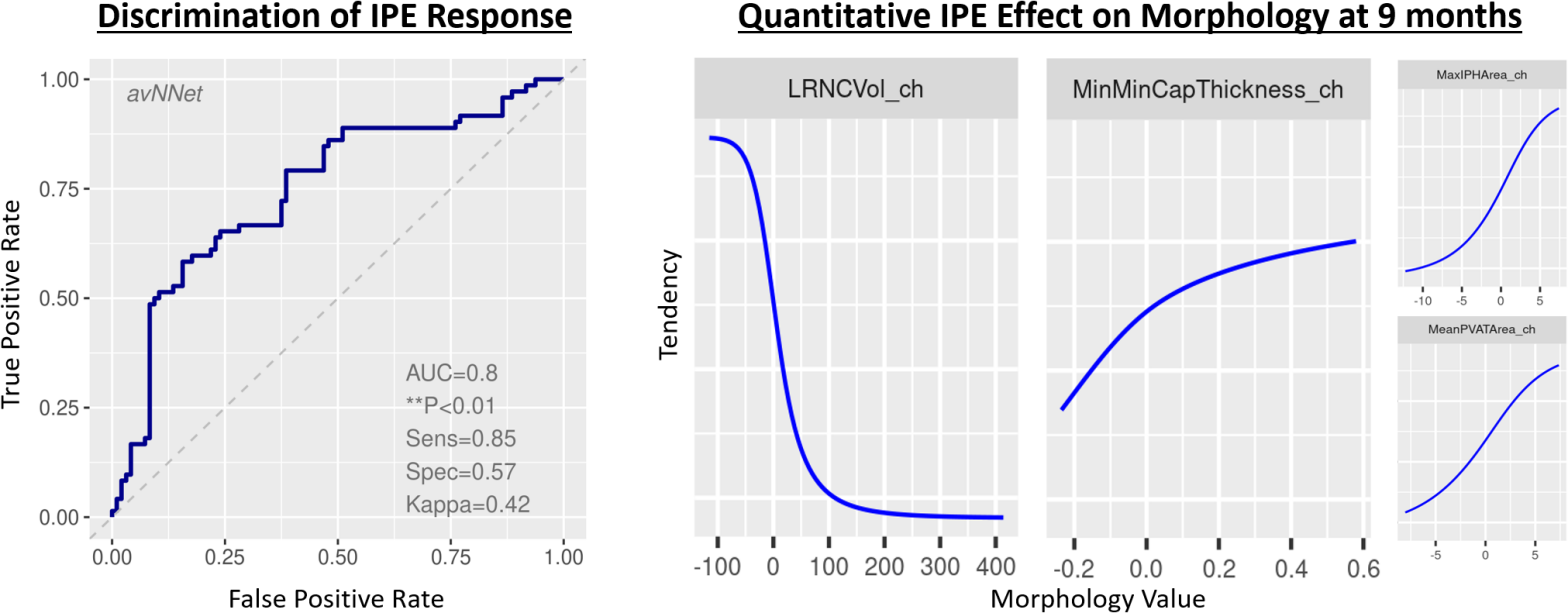
Classifier to infer patient representation that encodes meaningful changes in morphology. Model performance identifying relationships among quantitative plaque change features and drug response. LEFT: Receiver operating characteristic curve for the best-fit model on total coronary burden with quantitation formed by summing volumes, scaling proportions, and applying maxima, minima, and mean values across 3D regions of principal coronary arteries inclusive of anatomic and tissue characteristic regions. RIGHT: identification of feature tendencies stated in terms of probability of being on drug, where _ch suffix signifies change in the variable.

The best-performing multivariable model for the individual patient response was an averaged neural network (28) of a lipid-rich morphology. Each layer of the neural network produces a patient representation of the input patterns that is more abstract than the previous level because it is obtained by composing more non-linear operations. These models demonstrated a previously unreported ability to determine response on a per-patient individual basis by considering not only LRNC volume but using other biologically related morphological changes notably including wall and cap thickness that generalize to independent validation patients (Figure 5). The per-patient response assessment was possible using this optimized neural network model at the 9-month follow-up, *p* < 0.01. Table 3 presents the optimized neural network model that evaluated the change from baseline to 9 months. The model extrapolated to sequestered unseen patients using stratified portioning to form a validation set of patients to achieve an AUC of 0.74.

**Table 3:** Per-patient multivariable model performance.

Model type	Cross-validation performance					Sequestered hold-out performance			
	AUC	AUC *p*-value	Sens	Spec	Kappa	AUC	Sens	Spec	Kappa
avNNet	0.8 (0.72, 0.88)	*p* < 0.01	0.85 (0.79, 0.92)	0.57 (0.45, 0.7)	0.42 (0.27, 0.57)	0.74	0.60	0.88	0.49

## 4. Discussion

Cardiovascular disease, including stroke, peripheral artery disease, and coronary artery disease, is the most common cause of death and disability worldwide. Despite treatment with relatively low-cost statins to lower the low-density lipoprotein, high levels of residual risk remain, necessitating additional treatments. Hypertriglyceridemia possibly contributes to this residual risk. The REDUCE-IT established that IPE reduces CV risk in the patients with hypertriglyceridemia. Nonetheless, the CV benefit of IPE probably did not result primarily from triglyceride lowering (4). To gain further mechanistic insight into the benefits of IPE, this study evaluated plaque changes in response to IPE treatment compared with placebo.

The primary study analysis demonstrated that plaque burden decreases in the IPE-treated patients (9). The present study shows such changes in less time on drug and to do so at the individual level, findings not described in the primary study report. At the mid-way analysis at 9 months, LRNC differed significantly between IPE and placebo, alterations that continued for the 18-month duration of the study. This observation indicates that IPE has a measurable effect at 9 months, with that beneficial effect continuing for at least 18 months. This study found the histologically defined LRNC to be a superior measure as compared with LAP, which did not achieve significance at 9 months. LRNC is defined objectively, is less subject to variability than LAP assessment, and has stronger ties to the histological research basis, particularly given that necrosis is often not captured by the assessment of LAP and the inability to separate IPH without algorithms that account for tissue distributions. Moreover, the successful generation of predictive per-patient models captured the plaque characteristics. The per-patient multivariable models of characteristics that reliably classified the individual responses of the patients were demonstrated. The ability to assess response at the individual patient level with a high C-statistic demonstrates the utility of multivariable modeling to build on the cohort mean effects to capture the individual physiologic variability of the patients.

Specifically, the maximum wall thickness increased in the placebo arm and decreased in the IPE arm. The majority of the reduction in wall thickness was evident at 9 months but continued through 18 months. The fibrous cap thickness decreased in the placebo arm and increased in the IPE arm, a feature that may reflect plaques being less prone to rupture and provoke thrombosis. We conjecture that this is due to the regression of LRNC but could also be due to luminal surface changes that may warrant additional study. The change in LRNC increased among those on placebo and initially decreased among those on IPE, but then modestly grew from 9 to 18 months, which yielded a large net difference in favor of IPE. The wall thicknesses increased in both arms but less in the IPE arm at both 9 and 18 months. Given the wall thickness changes, IPE appears to improve uniformity, and features associated with more stable lesions. The morphological patient representation inferred from this supervised classification model could benefit other tasks, such as predicting adverse events.

**Figure 6:**
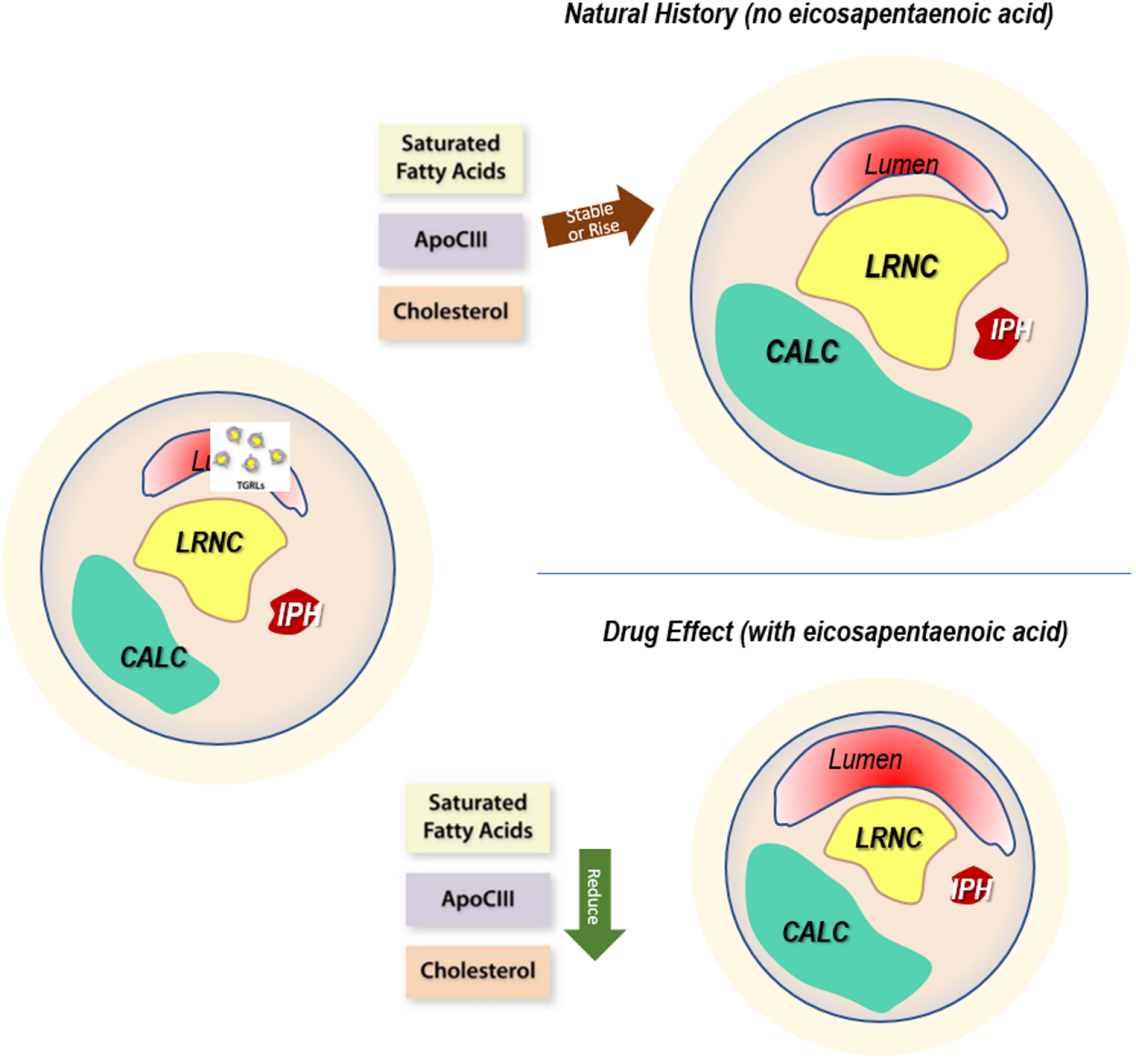
Drug effect. The mechanistic rationale for plaque changes based on drug action. Left: baseline characteristics of plaque burden show levels of stenosis, localized wall remodeling, calcification, lipid-rich necrotic core, and intraplaque hemorrhage. Upper right: without the administration of drug, the natural history of the plaque demonstrates increases associated with generally elevated levels of triglycerides, manifesting as high and sometimes increasing levels of saturated fatty acids, APOCIII, and cholesterol. This results in morphological measures and a proportional occupancy of low-attenuating components such as LRNC and IPH. In addition, the distance between lumen and LRNC, the cap thickness, decreases, with a net migration to less stable characteristics. Lower right: with icosapent ethyl, serum levels of these lipid species drop, causing reductions in features that contribute to instability, migrating the plaques to acquire more stable characteristics.

IPE improved multiple morphological characteristics associated with more stable lesions, and the patients on placebo developed less stable characteristics. Lesions with high-risk features (large necrotic core and thin cap) portend a greater likelihood of provoking future events (29–33) than plaques with larger LRNC and thinner fibrous caps (29–32, 34–37). Plaque morphology and composition may explain outcomes in lesions with normal and abnormal FFR (38, 39) and plaque rupture or erosion. Lesions with a large necrotic core may develop stenosis (38) due to reaching the limits of outward Glagovian remodeling, after which the lesions encroach on the lumen (40). Likewise, inflammatory insult or oxidative stress could result in local endothelial dysfunction (41–44). Abnormal endothelial vasomotor responses agree with the mechanistic understanding developed in (45) (Figure 6).

The primary outcome measure of this new analysis advances prior reported results in two important ways. First, the single-variable significance of LAP, as previously reported, was substantially improved with the more accurate analytic software and achieved significance at 9 months vs. 18 months. Second, the multivariable model made possible with the granularity of measurands for plaque characterization demonstrated statistical significance of the primary outcome measure (overall coronary burden *p* < 0.01).

The two secondary outcome measures of EVAPORATE pertaining to plaque included change in morphology and the composition of non-calcified coronary atherosclerotic plaque (NCP). The patients on IPE demonstrated large decreases in all three measures of wall remodeling (volume, area, and thickness), overall plaque burden, LRNC, and calcification by volume, maximum area, and proportional occupancy of the tissue type relative to the whole wall for the patients on IPE compared with the patients on placebo. The patients showed favorable increases in cap thickness on IPE compared with those on placebo. As plaques in the placebo group continued to expand despite maximum doses of statins, this study provides evidence that the LRNC reduction resulted solely from the IPE treatment.

Our study had limitations. First, this study is limited to EVAPORATE trial data, thus its generalization to other drugs and populations would require validation in other trials. This initial finding should encourage this undertaking. Second, the small sample size is a limitation of this study. Whereas the sample size was adequate as a feasibility study, larger cohorts should be studied to assess the generalizability of the results. Specifically, larger scale comparisons between this and other methods, as well as between different multivariable modeling approaches for individual patient response in larger cohorts, are warranted. In addition, while we quantified the link of drug effect on plaque, a confirmation that those plaque changes relate causally to event reduction warrants further study.

## 5. Conclusion

We build on the primary EVAPORATE analysis, which concluded that adding IPE to statin therapy yields quantitatively assessed changes in plaque morphology assessed by CTA that reached statistical significance at 18 months. This study used a software analytic technique that showed such changes in less time on drug (being significant at 9 months) and also demonstrated the feasibility of measuring such changes at the individual level, thereby extending the primary study results. This new analysis further demonstrated that the use of histologically defined terms and approaches allows for a more direct assessment of tissues consistent with reduced likelihood of plaque disruption and thrombosis. Specifically, IPE-induced decreases in LRNC and increases in cap thickness may mechanistically contribute to the reduction in clinical events shown in the REDUCE-IT. Using our technique, such effects can not only be measured at a cohort level, but also determined reliably for individual patients, which may be used as a response marker in clinical practice. These measurements further elucidate the mechanisms of IPE action by providing a quantitative window into LRNC growth, cap thickness modulation, and remodeling. These initial results should encourage larger studies, investigations with other drugs, and further validation of the use of individual patient models.

## Data Availability

The original contributions presented in the study are included in the article, further inquiries can be directed to the corresponding author.
